# Quantifying the neuropsychiatric symptoms in post-acute sequelae of COVID-19 (PASC) using the NIH Toolbox^**®**^ and PROMIS

**DOI:** 10.1515/nipt-2022-0010

**Published:** 2022-08-15

**Authors:** Meghann Ryan, Huajun Liang, Eleanor Wilson, Andrea Levine, Shyamasundaran Kottilil, Thomas Ernst, Linda Chang

**Affiliations:** Program in Neuroscience, University of Maryland School of Medicine, Baltimore, MD, USA; Department of Diagnostic Radiology and Nuclear Medicine, University of Maryland School of Medicine, Baltimore, MD, USA; Department of Diagnostic Radiology and Nuclear Medicine, University of Maryland School of Medicine, 670 W. Baltimore Street, HSF III, Baltimore, MD 21201, USA; Department of Medicine, Division of Infectious Disease, Institute of Human Virology, University of Maryland School of Medicine, Baltimore, MD, USA; Department of Medicine, Division of Pulmonary & Critical Care Medicine, University of Maryland School of Medicine, Baltimore, MD, USA; Department of Neurology, University of Maryland School of Medicine, Baltimore, MD, USA; Department of Neurology, Johns Hopkins University School of Medicine, Baltimore, MD, USA

**Keywords:** COVID-19, NIH toolbox, PASC, PROMIS

## Abstract

**Objective:**

To quantify neuropsychiatric symptoms reported by individuals with Post-Acute Sequelae of COVID-19 (PASC) using the NIH Toolbox for Assessment of Neurological and Behavioral Function (NIHTB) and Patient-Reported Outcomes Measurement Information System (PROMIS).

**Methods:**

30 PASC (20 women, 21–63 years) and 27 control (16 women, 25–68 years) participants completed three NIHTB batteries and selected PROMIS tests. Group differences on fully corrected T-scores were evaluated using analysis of covariance and Cohen’s *d* effect sizes. A linear regression model predicted the effects from time since diagnosis.

**Results:**

PASC had poorer emotional health and motor function than controls, including poorer locomotion, endurance and dexterity, but normal cognitive function, ∼7 months post-infection. PASC participants had a steeper age-related decline on the 2-Minute Walk Endurance Test than controls. T-scores on four cognitive and three motor tests improved with longer time since diagnosis.

**Conclusion:**

NIHTB and PROMIS captured the poorer emotional health and motor function in PASC, including the novel findings of deficits in locomotion and dexterity. The normal cognitive performance suggests subclinical effects that may be compensated by neural and cognitive reserves, and manifested subjectively by the negative psychological effects and fatigue. The persistent emotional and psychiatric symptoms necessitate mental health treatment be prioritized.

## Introduction

As of July 2022, more than a half-billion persons globally were infected with SARS-CoV-2; between 30 and 80% of survivors suffer from post-acute sequelae of COVID-19 (PASC), which may persist beyond one year [1]. Patients with PASC report neuropsychiatric symptoms such as fatigue, “brain fog”, memory problems, anxiety, and depression [1].

To assess these symptoms, we used the NIH Toolbox for Assessment of Neurological and Behavioral Function (NIHTB) and the Patient Reported Outcomes Measurement Information System (PROMIS) - two well-validated, computerized tools providing standardized assessments for clinical research [2, 3]. This study is the first to use three NIHTB batteries and selected tests from PROMIS to quantify the subjective neuropsychiatric symptoms in both hospitalized and non-hospitalized patients. We hypothesized that participants with PASC would have poorer emotional health, cognitive performance, and motor function compared to healthy controls.

## Results

### Participant demographics

The PASC group and controls had similar age, sex-proportion, Index of Social Position (ISP) [4], body mass index (BMI), years of education, race/ethnicity, and past-month alcohol, tobacco, and marijuana use (Table 1, all p>0.07).

**Table 1:** Participant characteristics (number, mean ± standard deviation or range).

	PASC, n=30	Controls, n=27	p-Value
Men/women	10/20	11/16	0.56^a^
Age (years, range)	42.7 ± 12.2 (21–63)	43.8 ± 12.2 (25–68)	0.73^b^
Body mass index	31.4 ± 8.8	28.0 ± 7.5	0.12^b^
Index of social position^d^	30.4 ± 13.8	28.9 ± 14.0	0.69^b^
Education			
Graduate/undergraduate/some college/high school	8/9/8/5	13/8/3/3	0.28^a^
*Race/ethnicity*			
White/Hispanic/Asian/Black/Biracial	19/1/0/9/1	9/4/2/12/0	0.07^a^
*History of substance use*			
Past month alcohol	24	22	0.95^a^
Lifetime alcohol	24	22	0.95^a^
Past month tobacco	1	1	1^c^
Lifetime tobacco	10	10	1^a^
Past month marijuana^e^	4	2	0.67^c^
Lifetime marijuana	15	6	0.03^a^
*Comorbid risk factors for COVID-19*			
Hypertension	6	4	0.26^c^
Diabetes	4	0	0.11^c^
Overweight/Obese	9/14	6/9	0.96^a^
COPD^f^	5	0	0.05^c^

Participants were recruited from the local community using online advertisements (Craigslist, Facebook Long-COVID support groups), flyers, word of mouth, and referrals from healthcare providers and post-COVID clinics. Phone screens were completed for 169 participants. Of those, 61 provided written informed consent and completed the in-person screening visit. Three were no longer eligible after the screening visit and one declined to continue participation. Inclusion criteria: (1) adults between 18 and 75 years; (2) ability to provide informed consent; (3) documented diagnosis of COVID-19 >6 weeks ago OR no history of COVID-19 with a negative antigen test at the screening visit. Exclusion criteria: (1) any confounding neurological, psychiatric, or medical disorders; (2) any current/past severe substance use disorder (Diagnostic and Statistical Manual of Mental Disorders 5), except for tobacco/cannabis; (3) pregnancy/breast-feeding. All participants underwent a structured medical history, physical/neurological examination, and urine toxicology screening. All provided written informed consent and the protocol was approved by the University of Maryland, Baltimore Institutional Review Board. Demographic data were compared between groups using ^a^Chi-Square test, ^b^ *t*-Test, or ^c^Fisher’s Exact Test-tests as appropriate. ^d^Index of Social Position (ISP) was calculated using the Hollingshead Four Factor Index of Socioeconomic Status [4]. ^e^Includes two PASC participants that used cannabidiol. ^f^Chronic Obstructive Pulmonary Disease.

### COVID-19 history and PASC symptoms

The average time since diagnosis was 219 ± 134 days, and 10 participants were hospitalized requiring supplemental oxygen therapy (Table 2). The most frequently reported PASC symptoms were concentration problems (89.7%), fatigue (82.8%), memory problems (79.3%), and depression/anxiety (69.0%).

**Table 2:** COVID-19 History (Number, Mean ± Standard Deviation or range).

COVID-19 history	PASC
Days since diagnosis [range]	219 ± 134 [42–484]
Hospitalized/non-hospitalized	10/20
*7-Point endpoint scale* ^a^	
1/2/3/4/5/6/7	0/2/5/3/0/0/20
*COVID-19 treatments*	
Low-flow/high-flow O_2_	6/3
Ventilator/ECMO	2/1
Steroid^b^/remdesivir	16/6
Monoclonal antibody^c^	3
Anticoagulant^d^/antibiotic^e^	1/5
*Current PASC symptoms*	%Total [% mild/% moderate/% severe]
Concentration problems	89.7 [24.1/51.7/13.8]
Fatigue	82.8 [44.8/27.6/10.3]
Memory problems	79.3 [20.7/37.9/20.7]
Depression or anxiety	69.0 [13.8/31.0/24.1]
Confusion	62.1 [6.9/13.8/41.4]
Sleep disturbances	62.1 [24.1/20.7/17.2]
Myalgia	58.6 [6.9/31.0/20.7]
Headaches	55.2 [13.8/34.5/6.9]
Dizziness	55.2 [13.8/13.8/27.6]
Gait disorder	51.7 [3.4/17.2/31.0]
Visual disturbances	48.3 [6.9/20.7/20.7]
Paresthesia	44.8 [13.8/13.8/17.2]
Lightheadedness	44.8 [10.3/17.2/17.2]
Coordination problems	41.4 [0.0/24.1/17.2]
Hyposmia	27.6 [3.4/10.3/13.8]
Dysgeusia	27.6 [6.9/6.9/13.8]
Urinary problems	24.1 [3.4/3.4/17.2]
Postural instability	13.8 [3.4/6.9/3.4]
Other neurological	13.8 [0.0/3.4/10.3]

All PASC participants provided a detailed COVID-19 history. All but one completed a PASC current symptom severity questionnaire. ^a^Acute COVID-19 severity was measured by the 7-Point Endpoint Scale [15]: (1) Death; (2) Hospitalized, requiring invasive mechanical ventilation/ECMO; (3) Hospitalized, requiring non-invasive ventilation/high flow oxygen; (4) Hospitalized, requiring low flow supplemental oxygen; (5) Hospitalized, not requiring supplemental oxygen, requiring ongoing medical care; (6) Hospitalized, not requiring supplemental oxygen, no longer requiring ongoing medical care; (7) Not Hospitalized. ^b^Dexamethasone, Prednisone, Methylprednisolone, or Hydrocortisone. ^c^Bamlanivimab or Etesevimab. ^d^Elliquis. ^e^Azithromycin or Ceftriaxone.

### NIHTB-Emotional Battery

**Figure 1:**
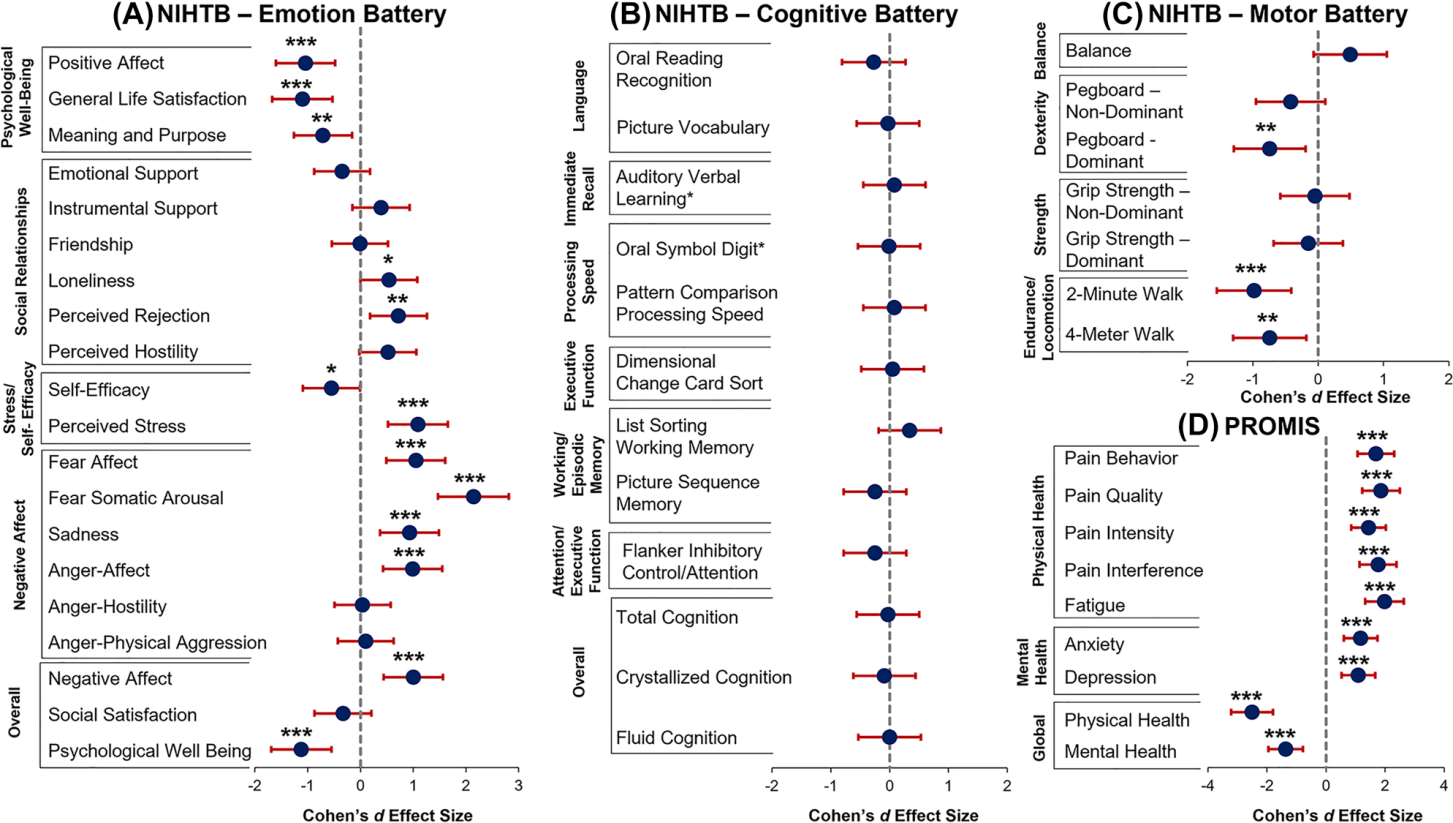
NIHTB and PROMIS captured the poorer emotional and motor health in PASC participants compared to controls. All NIHTB assessments were administered in person by trained researchers using an iPad. NIHTB emotional (NIHTB-EB), cognitive (NIHTB-CB), and motor (NIHTB-MB) batteries used the iPad NIH Toolbox App (v.1.23.4300) and PROMIS [3] surveys used REDCap. Domains and selected surveys are in the Supplement. All statistical analyses were performed using R (v.4.1.2). Analysis of covariance (ANCOVA), covaried for age, evaluated group (PASC vs. controls) differences on NIHTB and PROMIS T-Scores (corrected for age, sex, education, race/ethnicity) and Oral Symbol Digit and Auditory Verbal Learning raw scores. Cohen’s *d* effect sizes with 95% Confidence Intervals were calculated using the package ‘effsize’. For this exploratory study, significance was set at p<0.05. ^*^p≤0.05, ^**^p≤0.01, ^***^p≤0.001.

The PASC group had higher T-scores than controls for negative affect (Cohen’s *d*=1.00, 95% CI: [0.44, 1.56], p=4.4 × 10^−4^) and poorer psychological well-being (*d*=−1.12, 95% CI: [−0.55, −1.69], p=1.2 × 10^−4^) (Figure 1A, Table S1). Within the psychological well-being domain, the PASC participants had lower scores than controls for positive affect (*d*=−1.04, 95% CI: [−0.48, −1.61], p=2.6 × 10^−4^), general life satisfaction (*d*=−1.10, 95% CI: [−0.53, −1.67], p=1.6 × 10^−4^), and meaning and purpose (*d*=−0.71, 95% CI: [−0.16, −1.26], p=0.01). In the social relationship and stress/self-efficacy domains, PASC participants had higher scores than controls on perceived rejection (*d*=0.72, 95% CI: [0.17, 1.26], p=0.01) and stress (*d*=1.09, 95% CI: [0.52, 1.66], p=1.2 × 10^−4^) and lower self-efficacy (*d*=−0.55, 95% CI: [−0.01, −1.09], p=0.05). In the negative affect domain, compared to controls, PASC participants had more fear affect (*d*=1.05, 95% CI: [0.48 1.61], p=2.7 × 10^−4^), fear somatic arousal (*d*=2.14, 95% CI: [1.47, 2.81], p=1.2 × 10^−10^), sadness (*d*=0.93, 95% CI: [0.37, 1.49], p=0.001), and anger (*d*=0.99, 95% CI:[0.43, 1.55], p=0.001).

### NIHTB-Cognitive Battery

Despite the high prevalence of memory and concentration complaints by those with PASC, they had relatively normal performance on all cognitive tests, including working/episodic memory and attention/executive function, relative to the normative database (Table S1). They also performed similarly to the controls on all seven cognitive domains (Figure 1B, Table S1, all p>0.22).

### NIHTB-Motor Battery

In addition, endurance, assessed with the 2-Minute Walk Endurance Test, and locomotive abilities, assessed with the 4-Meter Walk Gait Speed, were lower in the PASC group than the controls (Figure 1C, Table S1, Cohen’s *d*=−0.98, 95% CI: [−0.41, −1.55], p=4.7 × 10^−4^; *d*=−0.74, 95% CI: [−0.18, −1.29], p=0.01, respectively). The PASC group also had poorer dominant hand dexterity than controls, assessed with the 9-Hole Pegboard Dexterity Test (*d*=−0.74, 95% CI: [−0.19, −1.29], p=0.01).

### PROMIS

The PASC group had significantly higher levels of depression, anxiety, fatigue, and pain measures (including pain interference, pain intensity, pain quality and pain behavior), which yielded poorer global mental and physical health T-scores than controls (Figure 1D, Table S1 all p<1.9 × 10^−4^).

### Assessment scores in relation to age, time since diagnosis, and illness severity

**Figure 2:**
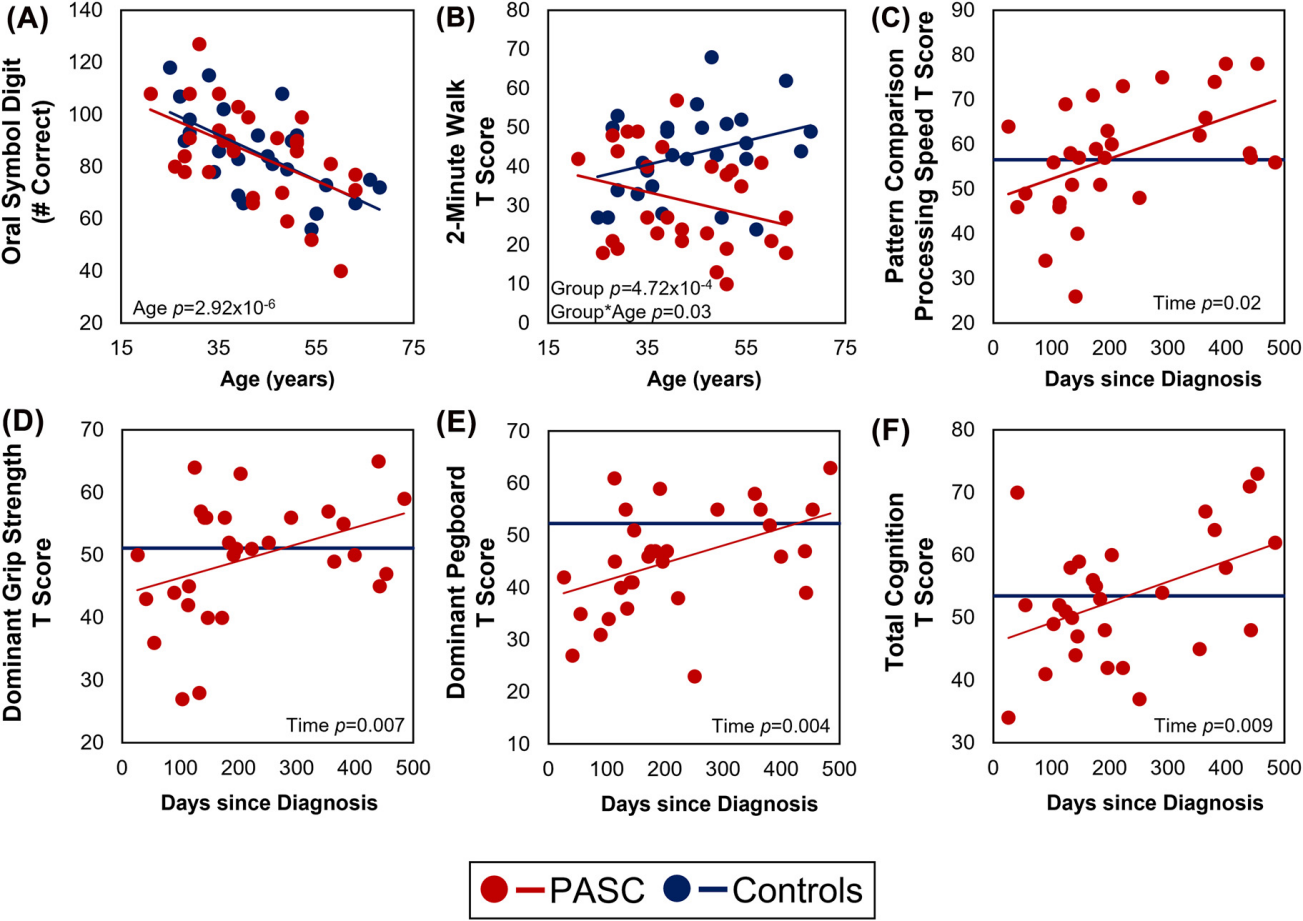
Assessment scores in Relation to age and time since diagnosis. A linear regression model used time since diagnosis to predict scores. The dark blue line in panels C–F indicates the average control T score for that assessment.

All participants showed age-related decline on the Oral Symbol Digit, Pattern Comparison Processing Speed, and Auditory Verbal Learning assessments (Figure 2A, Figure S1, all p<0.009). However, the PASC group showed steeper age-related decline on the 2-Minute Walk Endurance Test than the controls, primarily due to the lower T-scores in the older participants (Figure 2B, Age*Group p=0.03).

With longer duration since diagnosis of COVID-19, the scores were better on Pattern Comparison Processing Speed, Picture Vocabulary, Oral Reading Recognition, Total, Crystallized, and Fluid Cognition as well as Dominant/Non-Dominant Hand Grip Strength and Pegboard (Figure 2C–F, Figure S1, all p<0.04).

The hospitalized and non-hospitalized participants showed no group differences on any of the NIHTB or PROMIS measure (all p>0.1, Table S2)

## Discussion

This study is the first to quantify subjective neuropsychiatric PASC symptoms using NIHTB and PROMIS in hospitalized and non-hospitalized COVID-19 participants. Consistent with prior reports [5, 6], the NIHTB-EB captured poorer emotional health in PASC. However, our selected PROMIS measures assessed more domains than previous reports using the tool only in hospitalized patients [7, 8], by demonstrating that psychological effects, including depression and anxiety, are present even in our primarily outpatient participants.

The poorer endurance on the 2-Minute Walk Test in our PASC group is consistent with findings using the 6-Minute Walk Test [5] and Short Physical Performance Battery [9] in discharged COVID-19 patients 6 months after discharge. However, our assessment captured abnormalities in a mostly non-hospitalized, younger sample. We provided fully-corrected, standardized T-Scores (corrected for age, sex, race and education) for ease of comparison, and showed a steeper age-related decline than controls, suggesting even poorer endurance in the older participants with PASC. The slower performance on 4-Meter Walk Test and 9-Hole Pegboard Test (dominant hand) are novel findings suggesting COVID-19 might also impact locomotion and dexterity.

Our PASC participants reported a high prevalence of concentration/memory problems manifested as “brain fog”. However, multiple cognitive domains on the NIHTB-CB showed no cognitive deficits and they performed similarly to the uninfected controls. The cognitive impact of COVID-19 remains controversial in the literature. Our results align with another study that utilized a battery of neuropsychological tests to evaluate a comparable patient population with long COVID symptoms and found normal cognitive performance on the Montreal Cognitive Assessment (MoCA) [10]. However, another study using the MoCA reported poorer cognitive performance in a smaller sample [11] and self-reported Quality of Life assessments suggested cognitive impairment [6]. The NIHTB-CB is well-validated and highly sensitive, capable of detecting even mild cognitive impairment [12]. Additionally, the age-related declines in Oral Symbol Digit, Pattern Comparison Processing Speed, and Auditory Verbal Learning scores further validate the sensitivity of the Cognitive Battery by demonstrating known age-related declines in processing speed and immediate recall. Because of the normal cognitive performance on objective batteries despite the neurocognitive complaints, we hypothesize that these PASC participants may have subclinical effects that are compensated by neural and cognitive reserves, and possibly further exacerbated by the negative psychological effects and fatigue. Furthermore, an imbalance between GABAergic and dopaminergic activity in the cortex of COVID-19 survivors may contribute to fatigue and the subsequent subjective cognitive deficits [11]. The discrepancy between the subjective complaints and normal objective cognitive measures should be further evaluated using quantitative functional MRI to assess for increased usage of the brain reserves to compensate for the cognitive deficits and edited magnetic resonance spectroscopy to evaluate the brain GABA concentrations.

Consistent with a prior longitudinal study [13], better cognitive scores were associated with more remote COVID-19 diagnoses in the current study. Hence, the normal cognitive performance in participants with PASC might also result from normalization of prior deficits. Motor abilities showed a similar trend. However, similar to another convalescent COVID-19 sample [14], emotional health scores remained poor with time. Together, these results suggest a longer-lasting clinical impact on emotional health than cognition and motor function that necessitate further investigation. For example, evaluation of neurometabolite concentrations using magnetic resonance spectroscopy may determine whether the persistent psychiatric symptoms may be related to neuronal injury due to oxidative stress, and/or neuroinflammation.

This study is limited by its cross-sectional design, and the small subsample of hospitalized PASC participants. Furthermore, there exists the possibility of selection bias for the PASC group to include those with more severe symptoms. Knowledge of the PASC diagnosis may have also influenced the subjective reporting. Future studies should include a larger sample size, COVID-19 recovers with and without PASC, and longitudinal follow-up of the post-COVID-19 participants.

In summary, approximately 7 months after COVID-19, participants showed persistent abnormalities on emotional/physical health, endurance, locomotion, and dexterity, but relatively normal cognitive performance. Lower endurance appeared to be worse in the elderly with PASC. Although cognitive and motor abilities may improve with time, the long-lasting emotional and psychiatric symptoms necessitate that mental health treatments be prioritized. Lastly, given our novel findings of motor deficits, clinical examinations of COVID-19 survivors should include evaluation of locomotion and dexterity.

## Supplementary Material

Supplementary Material Details
