# Supplementary material for: Quantifying the neuropsychiatric symptoms in post-acute sequelae of COVID-19 (PASC) using the NIH Toolbox ® and PROMIS
Source: NeuroImmune Pharm Ther. 2022 Aug 15;2(2):95–101. doi: 10.1515/nipt-2022-0010 (PMC10373798; doi:10.1515/nipt-2022-0010)
Supplement: Supplementary file 1 — Supplementary Material Details [file j_nipt-2022-0010_suppl.docx]

**SUPPLEMENTAL FILE**

**Quantifying Neuropsychiatric Symptoms in Post-Acute Sequelae of COVID-19**

**using NIH Toolbox® and PROMIS**

Meghann C. Ryan MS^1^, Huajun Liang PhD^2^, Eleanor Wilson MD, MHS^3^, Andrea Levine MD^4^, Shyamasundaran Kottilil MD, PhD^3^, Thomas Ernst PhD^2,5^, and Linda Chang MD, MS^2,5,6^

1. Program in Neuroscience, University of Maryland School of Medicine, Baltimore, MD, USA
2. Diagnostic Radiology and Nuclear Medicine, and Neurology, University of Maryland School of Medicine, Baltimore, MD, USA
3. Institute of Human Virology, Department of Medicine, Division of Infectious Disease, University of Maryland School of Medicine, Baltimore, MD, USA
4. Department of Medicine, Division of Pulmonary & Critical Care Medicine, University of Maryland School of Medicine, Baltimore, MD, USA
5. Department of Neurology, University of Maryland School of Medicine, Baltimore, MD, USA
6. Department of Neurology, Johns Hopkins University School of Medicine, Baltimore, MD, USA

**Corresponding Author**

Linda Chang, MD, MS

670 W. Baltimore Street, HSF III

Baltimore, MD 21201, USA.

Phone: 410-706-1036

Email: [Linda.Chang@som.umaryland.edu](mailto:Linda.Chang@som.umaryland.edu)

**Supplemental Methods**

***NIH Toolbox and PROMIS***

The NIHTB Emotional Battery^1^ included the following domains: Psychological Well-Being (Positive Affect CAT Age 18+ v2.0, General Life Satisfaction CAT Age 18+ v2.0, Meaning and Purpose CAT Age 18+ v2.0), Social Relationships (Emotional Support FF Age 18+ v2.0, Instrumental Support FF Age 18+ v2.0, Friendship FF Age 18+ v2.0, Loneliness FF Age 18+ v2.0, Perceived Rejection FF Age 18+ v2.0, Perceived Hostility FF Age 18+ v2.0), Self-Efficacy and Stress (Self-Efficacy CAT Age 18+ v2.0, Perceived Stress FF Age 18+ v2.0), and Negative Affect (Fear Affect CAT Age 18+ v2.0, Fear Somatic Arousal FF Age 18+ v2.0, Sadness CAT Age 18+ v2.0, Anger-Affect CAT Age 18+ v2.0, Anger-Hostility FF Age 18+ v2.0, Anger-Physical Aggression FF Age 18+ v2.0).

The NIHTB Cognitive Battery^2^ assessed Attention/Executive Function (Flanker Inhibitory Control and Attention Test Age 12+ v2.1) Episodic Memory (Picture Sequence Memory Test Age 8+ Form A v2.1), Working Memory (List Sorting Working Memory Test Age 7+ v2.1), Language (Picture Vocabulary Test Age 3+ v2.1, Oral Reading Recognition Test Age 3+ v2.1), Executive Function (Dimensional Change Card Sort Test Age 12+ v2.1), Processing Speed (Pattern Comparison Processing Speed Test Age 7+ v2.1, Oral Symbol Digit Test Age 8+ v1.0), and Immediate Recall (Auditory Verbal Learning Test (Rey) Age 8+ v2.0).

The NIHTB Motor Battery^3^ evaluated locomotion (4-Meter Walk Gait Speed Test Age 7+ v2.0), endurance (2-Minute Walk Endurance Test Age 3+ v2.0), strength (Grip Strength Test Age 3+ v2.0), dexterity (9-Hole Pegboard Dexterity Test Age 3+ v2.0), and balance (Standing Balance Test Age 7+ v2.0).

The selected PROMIS^4^ surveys assessed Physical Health (Fatigue v1.0, Pain Intensity 3a v1.0, Pain Interference v1.1, Pain Quality v2.0, Pain Behavior v1.0), Mental Health (Anxiety v1.0, Depression v1.0), and Social Health (Global Social Activities/Roles). Global General Health, Mental Health, and Physical Health scores were calculated.

**Supplemental Table S1. Average Group T Scores for the NIH Toolbox and PROMIS**

| **NIHTB Emotional Battery** | **Assessment** | **Controls** | **PASC** | **Group**  **P Value** |
| --- | --- | --- | --- | --- |
| Psychological Well-Being | Positive Affect | 51.41±8.37 | 41.20±10.90 | **2.57x10^-4^** |
|  | General Life Satisfaction | 52.56±8.40 | 42.10±10.36 | **1.55x10^-4^** |
|  | Meaning and Purpose | 50.67±8.66 | 43.87±10.36 | **0.01** |
| Social Relationships | Emotional Support | 49.30±9.30 | 45.83±10.19 | 0.20 |
|  | Instrumental Support | 42.96±12.43 | 47.27±9.61 | 0.12 |
|  | Friendship | 46.56±8.28 | 46.40±12.80 | 0.96 |
|  | Loneliness | 52.70±9.77 | 58.40±11.19 | **0.05** |
|  | Perceived Rejection | 47.59±8.51 | 54.93±11.61 | **0.01** |
|  | Perceived Hostility | 45.52±10.89 | 50.67±9.09 | *0.07* |
| Self-Efficacy and Stress | Self-Efficacy | 51.44±8.72 | 46.67±8.64 | **0.05** |
|  | Perceived Stress | 46.07±10.99 | 58.20±11.16 | **1.18x10^-4^** |
| Negative Affect | Fear Affect | 48.67±12.70 | 60.60±10.09 | **2.73x10^-4^** |
|  | Fear Somatic Arousal | 44.44±8.13 | 66.40±11.84 | **1.21x10^-10^** |
|  | Sadness | 44.52±9.77 | 55.30±13.00 | **0.001** |
|  | Anger-Affect | 43.37±10.82 | 53.63±9.96 | **0.001** |
|  | Anger-Hostility | 51.26±11.91 | 51.70±11.31 | 0.95 |
|  | Anger-Physical Aggression | 48.48±8.97 | 49.33±8.04 | 0.73 |
| Overall Emotional Scores | Negative Affect | 46.11±11.10 | 57.27±11.19 | **4.42x10^-4^** |
|  | Social Satisfaction | 47.26±8.30 | 43.80±12.18 | 0.24 |
|  | Psychological Well-Being | 51.41±7.39 | 41.07±10.62 | **1.21x10^-4^** |
| **NIHTB Cognitive Battery** | | | | |
| Attention/Executive Function | Flanker Inhibitory Control and Attention Test | 47.26±11.21 | 44.53±10.50 | 0.38 |
| Episodic Memory | Picture Sequence Memory Test | 54.41±9.34 | 51.87±10.77 | 0.33 |
| Working Memory | List Sorting Working Memory Test | 49.33±9.45 | 52.40±8.79 | 0.22 |
| Language | Picture Vocabulary Test | 52.11±13.09 | 51.77±9.58 | 0.94 |
|  | Oral Reading Recognition Test | 54.70±7.19 | 52.67±8.03 | 0.34 |
| Executive Function | Dimensional Change Card Sort Test | 51.26±13.33 | 51.87±12.23 | 0.79 |
| Processing Speed | Pattern Comparison Processing Speed Test | 56.52±16.57 | 57.63±12.63 | 0.86 |
|  | Oral Symbol Digit Test * | 84.59±16.49 | 84.47±18.16 | 0.78 |
| Immediate Recall | Auditory Verbal Learning Test (Rey) * | 25.00±7.82 | 25.53±5.49 | 0.85 |
| Total Scores | Fluid Cognition | 52.41±12.82 | 52.37±10.74 | 0.99 |
|  | Crystallized Cognition | 53.63±9.99 | 52.76±8.95 | 0.77 |
|  | Total Cognition | 53.48±9.37 | 53.17±10.04 | 0.93 |
| **NIHTB Motor Battery** |  |  |  |  |
| Locomotion | 4-Meter Walk Gait Speed Test* | 1.34±0.33 | 1.11±0.28 | **0.01** |
| Endurance | 2-Minute Walk Endurance Test | 43.15±11.18 | 31.36±12.77 | **4.72x10^-4^** |
| Strength | Grip Strength - Dominant | 51.07±10.55 | 49.53±9.39 | 0.62 |
|  | Grip Strength - Nondominant | 49.04±11.31 | 48.47±10.06 | 0.91 |
| Dexterity | 9-Hole Pegboard Dexterity Test - Dominant | 52.33±8.81 | 45.37±10.00 | **0.01** |
|  | 9-Hole Pegboard Dexterity Test-Nondominant | 48.48±8.12 | 44.77±9.28 | 0.12 |
| Balance | Standing Balance Test | 41.48±13.18 | 46.89±8.28 | *0.09* |
| **PROMIS** | | | | |
| Mental Health | Depression | 45.72±6.71 | 54.71±9.46 | **1.90x10^-4^** |
|  | Anxiety | 47.26±8.34 | 58.06±9.97 | **5.39x10^-5^** |
| Physical Health | Fatigue | 40.03±7.95 | 57.21±9.27 | **1.22x10^-9^** |
|  | Pain Interference | 42.17±6.84 | 56.60±9.21 | **4.48x10^-9^** |
|  | Pain Intensity | 34.67±6.81 | 46.01±8.76 | **1.37x10^-6^** |
|  | Pain Quality | 33.27±6.77 | 47.42±8.28 | **3.59x10^-9^** |
|  | Pain Behavior | 41.48±8.71 | 55.66±8.05 | **3.14x10^-8^** |
| Overall Health Scores | Global Mental Health | 53.40±8.45 | 40.74±9.89 | **3.17x10^-6^** |
|  | Global Score Health Items | 57.42±9.14 | 36.65±7.42 | **6.08 x10^-13^** |

* Raw score. **Bold** indicates p≤0.05; *Italics* indicates 0.05<p≤0.1

**Supplemental Table S2. Hospitalized/Non-Hospitalized Average Scores**

| **NIHTB Emotional Battery** | **Assessment** | **Hospitalized** | **Non-Hospitalized** | **Group**  **P Value** |
| --- | --- | --- | --- | --- |
| Psychological Well-Being | Positive Affect | 45.20±14.37 | 39.20±8.41 | 0.10 |
|  | General Life Satisfaction | 44.30±8.41 | 41.00±11.24 | 0.46 |
|  | Meaning and Purpose | 41.60±11.22 | 45.00±10.01 | 0.41 |
| Social Relationships | Emotional Support | 47.30±7.56 | 45.10±11.40 | 0.52 |
|  | Instrumental Support | 49.60±7.88 | 46.10±10.36 | 0.33 |
|  | Friendship | 46.20±10.27 | 46.50±14.14 | 0.89 |
|  | Loneliness | 54.60±11.28 | 60.30±10.94 | 0.21 |
|  | Perceived Rejection | 51.10±11.79 | 56.85±11.32 | 0.26 |
|  | Perceived Hostility | 51.30±8.17 | 50.35±9.70 | 0.66 |
| Self-Efficacy and Stress | Self-Efficacy | 48.60±9.88 | 45.70±8.04 | 0.73 |
|  | Perceived Stress | 54.50±12.97 | 60.05±9.98 | 0.29 |
| Negative Affect | Fear Affect | 57.50±12.68 | 62.15±8.47 | 0.22 |
|  | Fear Somatic Arousal | 65.10±13.34 | 67.05±11.33 | 0.57 |
|  | Sadness | 51.30±16.01 | 57.30±11.14 | 0.23 |
|  | Anger-Affect | 52.30±12.45 | 54.30±8.75 | 0.53 |
|  | Anger-Hostility | 50.20±12.28 | 52.45±11.05 | 0.79 |
|  | Anger-Physical Aggression | 49.40±8.58 | 49.30±7.98 | 0.98 |
| Overall Emotional Scores | Negative Affect | 54.10±14.16 | 58.85±9.39 | 0.32 |
|  | Social Satisfaction | 46.60±10.81 | 42.40±12.84 | 0.37 |
|  | Psychological Well-Being | 42.70±12.37 | 40.25±9.88 | 0.51 |
| **NIHTB Cognitive Battery** | | | | |
| Attention/Executive Function | Flanker Inhibitory Control and Attention Test | 44.80±10.25 | 44.40±10.89 | 0.99 |
| Episodic Memory | Picture Sequence Memory Test | 56.40±11.71 | 49.60±9.79 | 0.21 |
| Working Memory | List Sorting Working Memory Test | 55.40±7.99 | 50.90±8.98 | 0.21 |
| Language | Picture Vocabulary Test | 51.60±7.43 | 51.85±10.67 | 0.98 |
|  | Oral Reading Recognition Test | 53.50±5.30 | 52.25±9.19 | 0.92 |
| Executive Function | Dimensional Change Card Sort Test | 52.00±11.09 | 51.80±13.04 | 0.77 |
| Processing Speed | Pattern Comparison Processing Speed Test | 51.70±12.49 | 60.60±11.90 | 0.14 |
|  | Oral Symbol Digit Test | 78.90±21.32 | 87.25±16.24 | 0.56 |
| Immediate Recall | Auditory Verbal Learning Test | 22.90±4.09 | 26.85±5.71 | 0.15 |
| Total Scores | Fluid Cognition | 52.90±10.98 | 52.10±10.89 | 0.93 |
|  | Crystallized Cognition | 52.80±4.32 | 52.74±10.73 | 0.89 |
|  | Total Cognition | 53.40±7.95 | 53.05±11.19 | 0.92 |
| **NIHTB Motor Battery** |  |  |  |  |
| Locomotion | 4-Meter Walk Gait Speed Test* | 1.11±0.40 | 1.11±0.21 | 0.73 |
| Endurance | 2-Minute Walk Endurance Test | 25.70±9.20 | 34.50±13.60 | 0.14 |
| Strength | Grip Strength - Dominant | 47.40±10.88 | 50.60±8.67 | 0.17 |
|  | Grip Strength - Nondominant | 47.90±12.64 | 48.75±8.85 | 0.44 |
| Dexterity | 9-Hole Pegboard Dexterity Test - Dominant | 43.70±8.54 | 46.20±10.77 | 0.37 |
|  | 9-Hole Pegboard Dexterity Test-Nondominant | 40.90±7.92 | 46.70±9.49 | 0.13 |
| Balance | Standing Balance Test | 49.10±5.67 | 45.59±9.40 | 0.22 |
| **PROMIS** | | | | |
| Mental Health | Depression | 50.92±10.76 | 56.61±8.40 | 0.14 |
|  | Anxiety | 55.39±11.71 | 59.40±9.00 | 0.33 |
| Physical Health | Fatigue | 53.24±11.29 | 59.20±7.64 | 0.12 |
|  | Pain Interference | 57.04±10.74 | 56.38±8.64 | 0.77 |
|  | Pain Intensity | 45.82±10.21 | 46.11±8.23 | 0.78 |
|  | Pain Quality | 46.13±11.08 | 48.07±6.72 | 0.44 |
|  | Pain Behavior | 55.58±9.29 | 55.70±7.62 | 0.71 |
| Overall Health Scores | Global Mental Health | 42.07±11.32 | 40.08±9.35 | 0.51 |
|  | Global Score Health Items | 36.40±8.20 | 36.78±7.22 | 0.87 |

Analysis of covariance (ANCOVA), covaried for age, evaluated group (hospitalized vs. non-hospitalized participants) differences on NIHTB and PROMIS T-Scores (corrected for age, sex, education, race/ethnicity) and Oral Symbol Digit and Auditory Verbal Learning raw scores.


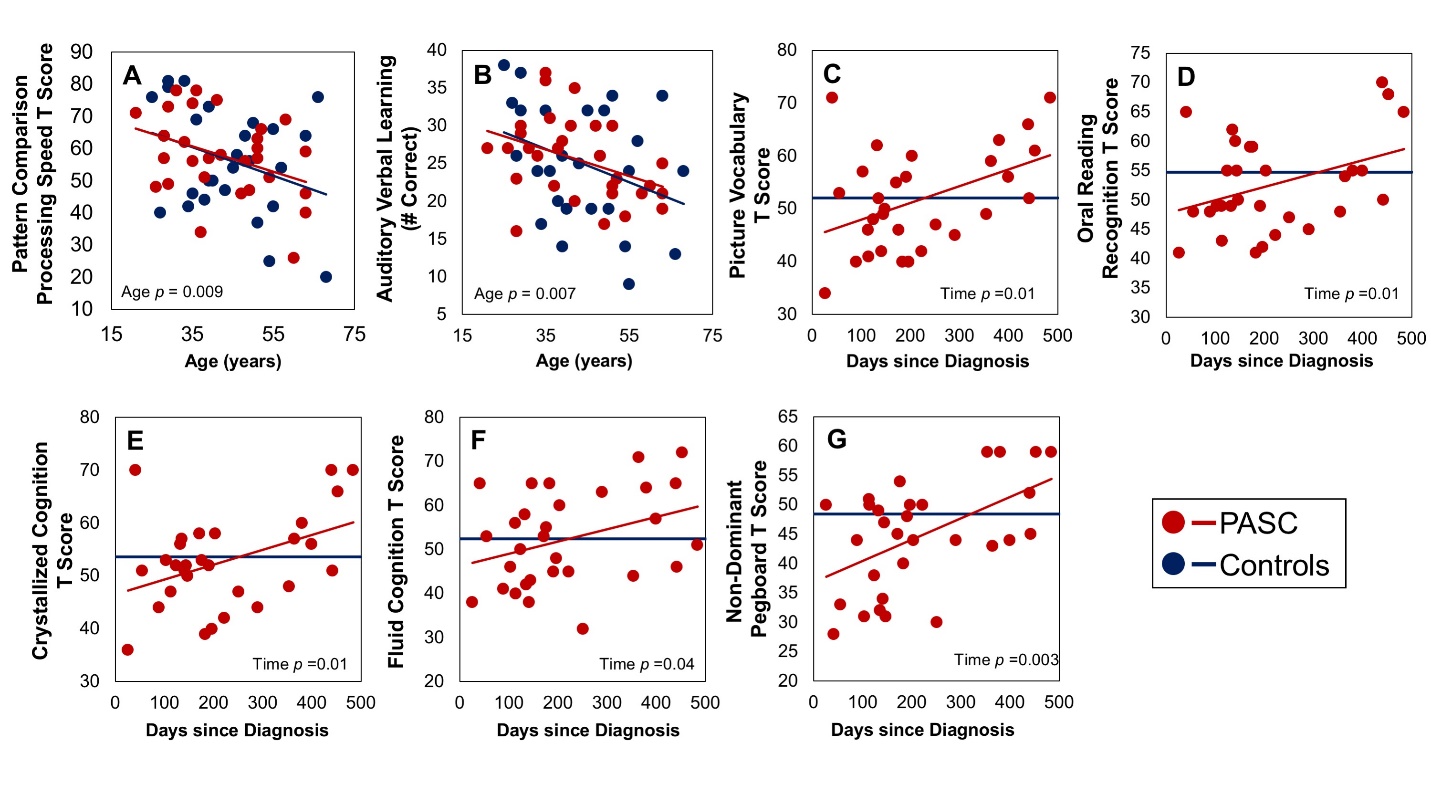


**Supplemental Figure S1. Assessment Scores in Relation to Age and Time Since Diagnosis.** In panels B-F, navy horizonal line indicates average control T scores.

**References**

1. Salsman JM, Butt Z, Pilkonis PA, et al. Emotion assessment using the NIH Toolbox. *Neurology.* 2013;80(11 Suppl 3):S76-86.

2. Weintraub S, Dikmen SS, Heaton RK, et al. Cognition assessment using the NIH Toolbox. *Neurology.* 2013;80(11 Suppl 3):S54-64.

3. Reuben DB, Magasi S, McCreath HE, et al. Motor assessment using the NIH Toolbox. *Neurology.* 2013;80(11 Suppl 3):S65-75.

4. Cella D, Riley W, Stone A, et al. The Patient-Reported Outcomes Measurement Information System (PROMIS) developed and tested its first wave of adult self-reported health outcome item banks: 2005-2008. *J Clin Epidemiol.* 2010;63(11):1179-1194.
